# Molecular Confirmation of *Taenia solium* Taeniasis in Child, Timor-Leste

**DOI:** 10.3201/eid3009.240238

**Published:** 2024-09

**Authors:** Hanna Jin, Sung-Tae Hong, Merita Antonio Armindo Monteiro, Endang da Silva, Odete da Silva Viegas, Felix dos Santos Lopes, Dong Hee Kim, Sung Hye Kim

**Affiliations:** Seoul National University, Seoul, South Korea (H. Jin, S.-T. Hong, D.H. Kim);; Ministry of Health, Dili, Timor-Leste (M.A.A. Monteiro, E. da Silva, O. da Silva Viegas);; World Health Organization Timor-Leste Country Office, Dili (F. dos Santos Lopes);; Hanyang University College of Medicine, Seoul (S.H. Kim)

**Keywords:** *Taenia solium*, human taeniasis, tapeworm, cysticercosis, *cox-1* gene, enteric infections, parasites, zoonoses, Timor-Leste

## Abstract

We report a case of *Taenia solium* taeniasis in a 10-year-old child in Timor-Leste, confirmed by molecular analysis, suggesting *T. solium* transmission to humans is occurring in Timor-Leste. Proactive measures are needed to improve public understanding of prevalence, geographic spread, and health implications of human taeniasis and cysticercosis in Timor-Leste.

The pork tapeworm, *Taenia solium*, causes human taeniasis and cysticercosis, which are considerable health problems in many developing countries (*1*). In Southeast Asia, *T. solium* infections are considered endemic, but epidemiologic data remain scarce (*2*). We report a case of *T. solium* taeniasis in Timor-Leste, confirmed by molecular methods.

In March 2019, as part of routine monitoring by the Timor-Leste Ministry of Health’s national control program targeting soil-transmitted helminthiasis, in collaboration with the World Health Organization’s country office, 1,121 fecal samples from school children in Timor-Leste were examined by using the Kato-Katz method. *Taenia* spp. eggs were identified in 4 samples. Subsequently, we conducted home visits for each affected child and administered a single dose of 10 mg/kg praziquantel (Shin Poong Pharmaceutical Co. Ltd, https://shinpoong.co.kr). We were able to collect expelled worm segments on the same day of treatment from a 10-year-old girl residing in Dili, the capital of Timor-Leste. Throughout most of her life, the child had remained in good health and had not manifested symptoms indicative of human taeniasis. Also, she had not traveled outside of the country.

The retrieved worm segments exhibited a flat, creamy white appearance, aligning with the typical macroscopic characteristics associated with *Taenia* spp. (Figure 1). Microscopic analysis of the segments revealed ≈50 gravid, 20 mature, and 20 immature proglottids of *T. solium*.

**Figure 1:**
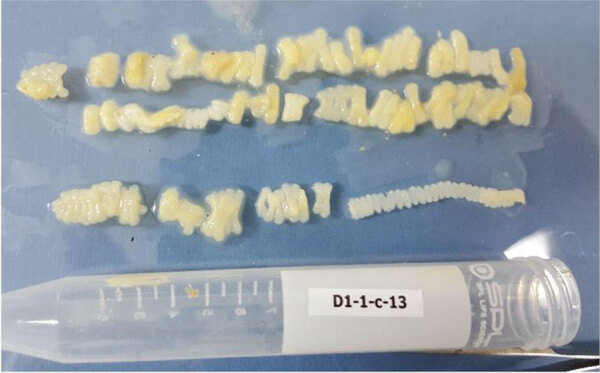
Proglottids of *Taenia solium* collected from a patient in Dili, Timor-Leste, in case study of molecular confirmation of taeniasis in a child. We collected the expelled worm segments from a 10-year-old girl on the same day she was treated with 10 mg/kg praziquantel.

To determine the species through molecular analysis, we isolated genomic DNA from 1 segment by using the DNeasy Blood & Tissue Kit (QIAGEN, https://www.qiagen.com), according to the manufacturer’s instructions. We performed PCR of genomic DNA to detect the parasite mitochondrial *cox-1* gene that encodes cytochrome c oxidase subunit I (Appendix) (*3*). We purified the PCR products by using DNA Clean & Concentrator-5 (Zymo Research, https://www.zymoresearch.com), according to the manufacturer’s protocol. Sanger sequencing was subsequently performed by Bioneer Co., Ltd. (https://www.bioneer.co.kr), which used an ABI3730XL instrument (Applied Biosystems/Thermo Fisher Scientific, https://www.thermofisher.com). We deposited the derived sequence in GenBank (accession no. PP837933.1) and compared it with other *cox-1* sequences in GenBank by using BLAST (https://blast.ncbi.nlm.nih.gov). The sequence showed 98.85%–100% identity with the *T. solium* mitochondrial *cox-1* gene. We used *cox-1* sequences for phylogenetic reconstruction (Figure 2; Appendix). We aligned DNA sequences by using ClustalW (http://www.clustal.org) and conducted evolutionary analyses by using MEGA11 (*4*). The sequence isolated in this study was shown to be most homologous with an isolate from Tulear (also known as Toliara), Madagascar (GenBank accession no. FM958316.1) (*5*). Consequently, molecular evaluation confirmed the infection was caused by *T. solium*.

**Figure 2:**
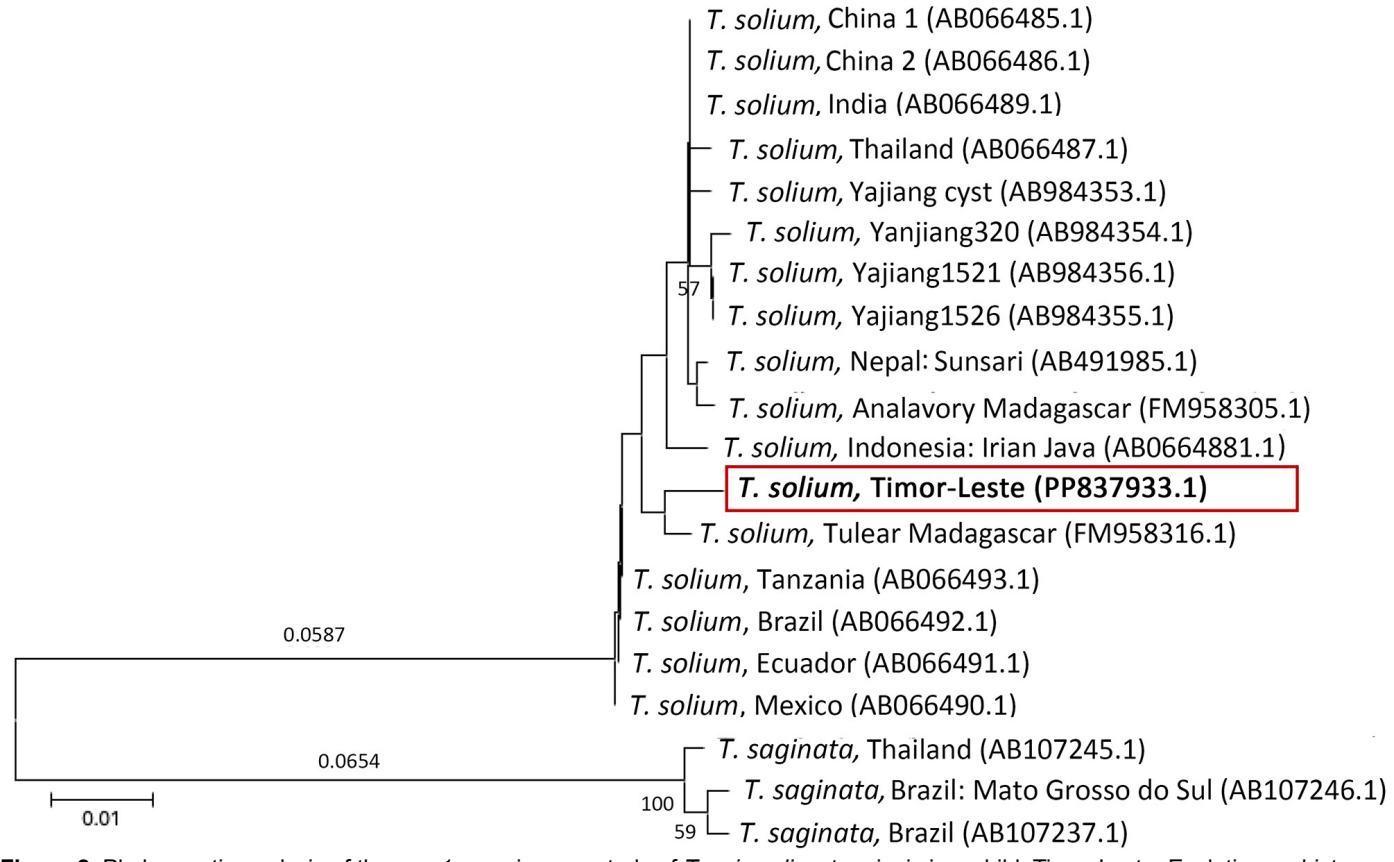
Phylogenetic analysis of the *cox-1* gene in case study of *Taenia solium* taeniasis in a child, Timor-Leste. Evolutionary history was inferred by using the neighbor-joining method and analysis was conducted by using MEGA11 (*4*). Red box and bold text indicates the sequence from this study. GenBank accession numbers are indicated in parentheses. Percentages of replicate trees in which the associated taxa clustered together in the bootstrap test (1,000 replicates) are shown below the branches. Tree is drawn to scale; branch lengths (above the branches) are in the same units as those of the evolutionary distances used to infer the phylogenetic tree. The evolutionary distances were computed by using the Kimura 2–parameter method. Analysis involved 20-nt sequences. Codon positions included were first + second + third + noncoding. All ambiguous positions were removed for each sequence pair (pairwise deletion option). A total of 480 positions were in the final dataset. Scale bar indicates nucleotide substitutions per site.

We report documented human *T. solium* taeniasis in Timor-Leste, an area where previous records of the parasite have been nearly absent (*6*). Clinically diagnosed neurocysticercosis in persons from Timor have been reported in Australia and Indonesia, suggesting the presence of *T. solium* in Timor-Leste (*7*,*8*). However, the only documentation of human taeniasis/cysticercosis within Timor-Leste is a case of oral cysticercosis in a person originally from Timor-Leste reported in Northern Ireland in 2015 (*9*). That particular patient exhibited symptoms of oral submucosal swelling and had relocated from Timor-Leste in 2006. Because no alternative sources of cysticercosis were identified, it is likely that the patient acquired the infection in Timor-Leste before migrating to Northern Ireland, a region where cysticercosis is not endemic (*9*). Similarly, a high probability exists that the child’s infection in this case study originated within Timor-Leste, because she had not traveled outside of the country before the worm was detected. Through interviews, we found that she had regular interactions with confined pigs in her backyard and with free-ranging pigs within the village where she lived previously. However, the presence of *T. solium* cysticerci in those pigs and potential infection status remains undetermined.

The *cox-1* sequence from the worm isolated in Timor-Leste was closely related to sequences collected in Toliara in southern Madagascar. According to a previous study conducted in Madagascar, specimens from Toliara had diverged from parasites of the African/South American genotype (*5*). However, the lack of data limits what we can infer about *T. solium* in Timor-Leste. Further epidemiologic studies are needed to determine the extent of *T. solium* infection in pigs and to guide the implementation of control programs.

In conclusion, *T. solium* infections have been identified as endemic in Timor-Leste, a nation previously devoid of documented cases. Considering the widespread practice of backyard pig farming and the presence of free-roaming pigs across much of the country (*10*), veterinarians and clinicians should be vigilant in suspecting this emerging zoonotic parasite as a cause of taeniasis, not only in pig populations but also in humans. Furthermore, we urge health authorities in Timor-Leste to take proactive measures to enhance public understanding of the prevalence, geographic spread, and health implications of human taeniasis and cysticercosis within the nation.

Appendix Additional information for molecular confirmation of *Taenia solium* taeniasis in child, Timor-Leste.
