# Supplementary material for: Molecular Confirmation of Taenia solium Taeniasis in Child, Timor-Leste
Source: Emerg Infect Dis. 2024 Sep;30(9):1964–7. doi: 10.3201/eid3009.240238 (PMC11346990; doi:10.3201/eid3009.240238)
Supplement: Appendix — Additional information for molecular confirmation of Taenia solium taeniasis in child, Timor-Leste. [file 24-0238-Techapp-s1.pdf]

Article DOI: https://doi.org/10.3201/eid3009.240238

*EID cannot ensure accessibility for supplementary materials supplied by authors. Readers who have difficulty accessing supplementary content should contact the authors for assistance.*

# Molecular Confirmation of *Taenia solium* Taeniasis in Child, Timor-Leste

## Appendix

## Additional Methods and Data

### PCR

For molecular confirmation, we amplified a 491 bp fragment of the *Taenia solium cox-1* gene (*1*). The PCR primers used were T1F (5′-ATA TTT ACT TTA GAT CAT AAG CGG-3′) and T1R (5′-ACG AGA AAA TAT ATT AGT CAT AAA-3′) (*2*).

We used AccuPower Hotstart PCR PreMix tubes (Bioneer Co., Ltd, https://www.bioneer.co.kr) for PCR. The reaction mixture contained 1 μL of template DNA (100 ng/μL) and 10 μmol/L of each primer, and diethyl pyrocarbonate–treated water was added to reach a total volume of 20 μL. PCR entailed an initial denaturation phase at 94°C for 3 minutes, succeeded by 40 cycles of denaturation (94°C for 1 minute), annealing (52°C for 1 minute), and extension (72°C for 1 minute), culminating in a final extension at 72°C for 10 minutes.

### Phylogenetic analysis

The evolutionary history was inferred by using the neighbor-joining method (*3*). The percentages of replicate trees in which the associated taxa clustered together in the bootstrap test (1,000 replicates) were calculated (*4*). The tree was drawn to scale; branch lengths were in the same units as those of the evolutionary distances used to infer the phylogenetic tree. The evolutionary distances were computed by using the Kimura 2–parameter method (*5*) and are designated as the number of base substitutions per site. The analysis involved 20-nt sequences. Codon positions were first + second + third + noncoding. All ambiguous positions were removed for each sequence pair (pairwise deletion option). A total of 480 positions were in the final dataset. Evolutionary analyses were conducted by using MEGA11 (*6*).

## Sequence Amplified by Using *Taenia* spp. *cox-1* Restriction Fragment Length Polymorphism–PCR Primers

GCGTCATTAACTTTGTTGGGTTTGTGGTCAGGTTTTGTAGGTTTAAGTTTTAGT
TTATTAATTCGTGTTAATTTTTTAGAACCTTATTATAATGTGATTTCTTTGGATTGTTA
TAAATTTTTGATTACTAATCATGGTATAATAATGATTTTCTTTTTTTTAATGCCTATTT
TAATTGGAGGTTTTGGTAAATACTTAATTCCATTGATAGGAGGATTATCGGATTTGA
ATTTACCACGTTTGAATGCTTTAAGTGCATGGTTGTTAGTTCCATCAATTGTTTTTCTT
TTAATTAGGATGTGTTTAGGCGCTGGTATAGGGTGGACTTTTTACCCACCTTTATCAT
CTTCGTTGTTTTCAAGAGGAAGTGGGGTAGATTTTTTAATGTTTTCTTTACATTTAGC
TGGTGTATCAAGTATTTTTAGTTCTATTAATTTTATATGTACATTATATAGAGTTTTTA
TGACTAATAATTTTTTCTCGTA

## References


1. Cho J, Jung BK, Lim H, Kim MJ, Yooyen T, Lee D, et al. Four cases of *Taenia saginata* infection with an analysis of COX1 gene. Korean J Parasitol. 2014;52:79–83. PubMed https://doi.org/10.3347/kjp.2014.52.1.79

2. Zarlenga DS, McManus DP, Fan PC, Cross JH. Characterization and detection of a newly described Asian taeniid using cloned ribosomal DNA fragments and sequence amplification by the polymerase chain reaction. Exp Parasitol. 1991;72:174–83. PubMed https://doi.org/10.1016/0014-4894(91)90135-J

3. Saitou N, Nei M. The neighbor-joining method: a new method for reconstructing phylogenetic trees. Mol Biol Evol. 1987;4:406–25. PubMed https://doi.org/10.1093/oxfordjournals.molbev.a040454

4. Felsenstein J. Confidence limits on phylogenies: an approach using the bootstrap. Evolution. 1985;39:783–91. PubMed https://doi.org/10.1111/j.1558-5646.1985.tb00420.x

5. Kimura D, Uga S. Simple and effective method for observation of suspended parasite specimens using vaseline and paraffin mixture. Parasitol Int. 2016;65:596–8. PubMed https://doi.org/10.1016/j.parint.2016.05.008

6. Tamura K, Stecher G, Kumar S. MEGA11: molecular evolutionary genetics analysis version 11. Mol Biol Evol. 2021;38:3022–7. PubMed https://doi.org/10.1093/molbev/msab120
